# Keeping Healthy in Your Skin—Plants and Fungi Used by Indigenous Himalayan Communities to Treat Dermatological Ailments

**DOI:** 10.3390/plants12071575

**Published:** 2023-04-06

**Authors:** Shiekh Marifatul Haq, Aadil Abdullah Khoja, Fayaz A. Lone, Muhammad Waheed, Rainer W. Bussmann, Ryan Casini, Eman A. Mahmoud, Hosam O. Elansary

**Affiliations:** 1 Department of Ethnobotany, Institute of Botany, Ilia State University, Tbilisi 0162, Georgia; 2 Department of Botany, Glocal University Saharanpur, Saharanpur 247121, India; 3 Department of Botany, Government Degree College (Women), Kupwara 193222, India; 4 Department of Botany, University of Okara, Okara 56300, Pakistan; 5 Department of Botany, State Museum of Natural History, Erbprinzenstrasse 14, 76133 Karlsruhe, Germany; 6 School of Public Health, University of California, Berkeley, 2121 Berkeley Way, Berkeley, CA 94704, USA; 7 Department of Food Industries, Faculty of Agriculture, Damietta University, Damietta 34511, Egypt; 8 Department of Plant Production, College of Food & Agriculture Sciences, King Saud University, P.O. Box 2460, Riyadh 11451, Saudi Arabia

**Keywords:** dermatology, ethnomedicine, wound healing, traditional knowledge

## Abstract

Dermatological ailments are a major health problem, especially when related to human immune deficiency syndrome and acquired immune deficiency. The goal of this study was to identify the medicinal plants used by the indigenous peoples of the Northwestern Himalayas to treat dermatological diseases. Several field trips were conducted in the spring and summer seasons of 2020–2021 to collect the plants of dermatological value and information about their use through open-ended semi-structured interviews (*n* = 53) and group discussions (*n* = 33). The current investigation found 64 ethnomedicinal plants belonging to 34 families commonly used to treat a variety of dermatological ailments. The main growth form was herbs (80%), followed by trees (8%) and ferns (6%). It was found that leaves (51%) were the most commonly used plant part, followed by roots and the whole plant. Wound healing was the most dominant application, with 18 plant species used, followed by skin burns cured by 11 plant species and skin boils by eight plant species. Out of the total (18%) of medicinal plants with cosmetic uses, i.e., roots of *Jurinea dolomiaea*, *Rheum webbianum*, and *Rheum spiciforme* were crushed into powder and mixed with turmeric, and the paste is applied topically for glowing skin. Among the various preparation methods, paste (38%) was the most common way of preparation, followed by poultice (29%) and infusion (9%). Between ethnic groups, the maximum homogeneity was between Gujjar and Bakarwal ethnic groups (23 species, 36%), followed by Gujjars and Kashmiri (14 species, 22%). Bakarwals and Gujjar people live in the same geographical location, and they graze their animals in pastures, practice extensive transhumance pastoralism, and pass through different ecological landscapes, thus having sufficient experiences with certain plants and retaining more knowledge. The species identified with the highest utilization based on the number of citations and use value included *Ficus carica*, *Cichorium intybus*, *Euphorbia wallichii*, *Pinus wallichiana*, *Plantago major*, *Jurinea dolomiaea*, and *Artemisia absinthium*. The findings of this study demonstrate that people who reside in the Northwestern Himalayas region still rely on medicinal plants.

## 1. Introduction

Ethnodermatology is a branch of Ethnobiology that deals with the identification and management of skin conditions and infections, as well as skincare and aesthetics in ethnically diverse populations [1]. The serious systemic diseases that develop as complex and severe damage from burns or generalized dermatitis demonstrate how important the skin is to the overall physiology of the body [1]. The skin barrier helps to prevent microbial infections, limit environmental contaminant absorption, and passive water loss from the body [2]. Natural residents derived from plants have a strong potential for cetaceous protection [3]. Despite the fact that plants have been used for skin health care management in many parts of the world since ancient times, herbal products were neglected for a long time after the development of modern synthetic soaps, creams, and other cosmetics [4,5]. However, as time progressed, the side effects of these chemical cosmetics became apparent, and people started turning back to herbal products for skin health care management. Traditional herbal medicine research became widely accepted around the world in the latter half of the 20th century as a result of the discovery of medicinal plants from native pharmacopeias that have been demonstrated to have significant healing power, either in their natural state or as a source of new pharmaceuticals, and the recognition that herbal formulations are less toxic than most pharmaceutical agents [6].

Traditional herbal practitioners play an important role in health care, especially in developing countries. Global estimates indicate that over 75% of the world’s population cannot afford products of the Western pharmaceutical industry and depend on the use of traditional medicines mainly obtained from plants [7].

The skin is an external organ covering the body and serves many important functions, including percutaneous absorption, organ protection, fluid preservation, body shape maintenance, temperature regulation, and eliminating toxins from the body by sweat excretion [8]. The etiology of skin diseases displays a close connection between an individual’s health and the sociocultural environment [9]. Skin diseases affect people of all age groups and gender. Ethno-medicinal studies showed that herbal medicine is an alternative therapy for the treatment and control of skin ailments. Skin ailments such as boils, itching, ringworm, skin disorders, leprosy, wound, dermatitis, eczema, scabies, skin allergy swelling, and psoriasis are caused by a variety of microorganisms [10]. Previous reports found that wound healing, eczema, dermatitis, fungal diseases, pyoderma, scabies, and skin allergies are the largest group of skin diseases that occur in most countries [7]. Most plants used for treating skin disorders may have other additional properties, such as anti-inflammatory, anti-microbial, anti-viral, cicatrizing, hemostatic, and analgesic effects requiring pharmacological confirmation [8].

Due to its remarkable biological and cultural diversity, the Northwestern Himalaya area is one of the most significant global repositories of medicinal plants and associated traditional knowledge. In terms of species composition and richness, the vegetation of the western and northern Himalayas exhibit increasing similarity to that of the Hindu Kush and the monsoon belt of the Karakorum mountains, respectively, due to comparable geological, physiographic, and climatic conditions [11]. At higher altitudes, especially in alpine and subalpine habitats, the limiting factor of altitude further reinforces the floristic similarities between the higher-elevation vegetation of the Hindu Kush and the Western Himalayas [12]. In the secluded valleys of the Himalayas, local communities rely on natural resources, including plants, for their livelihoods, often migrating with their livestock to summer pastures in accordance with the changing seasons. They possess valuable knowledge regarding ecosystem services [13]. However, the intensive use of plants for medicinal purposes, grazing, and fodder has resulted in heightened pressure on plant biodiversity, thereby posing significant implications for the long-term sustainability of the ecosystem. This continuous pressure has caused some species to become endangered, and they are at risk of extinction in the near future. The conflict between anthropogenic activities and biodiversity is apparent, as people tend to prioritize their own needs and place pressure on rare species [14].

In many areas where herbal medicine was developed and supported by oral tradition, its use has recently decreased. This could lead to the loss of important knowledge about the plants that will be used in the future [15]. Due to their lack of side effects, herbal medicines are becoming increasingly popular. Additionally, as healthcare costs rise, consumer interest in inexpensive treatments has encouraged them to reconsider the potential of alternatives [16]. The aim of the current research was to contribute to an improved understanding of wild ethnomedicinal plant uses to treat dermatological disorders from the remote area (administrative district “Kupwara”) of Kashmir Himalayas. The main objectives of the study are (a) the collection of the medicinal plants used in the treatment of dermatological disorders; (b) to enlist the administration of different disease categories; (c) to record the medicinal plants used to treat dermatological disorders during the COVID-19 pandemic.

## 2. Results and Discussion

### 2.1. Demographic Status of Informants

A total of 86 informants were interviewed, all of whom followed Islam. Among them, male respondents had more knowledge about medicinal plants than female informants. Of the 86 informants, 58 were men, and the remaining 28 were women informants (Table 1). Most of the selected interviewees were illiterate, and a few had higher education. The informants were selected using snowball sampling based on their traditional knowledge about the use of medicinal plants to cure various diseases. In order to ensure the validity of traditional knowledge, an ongoing relationship with the local residents was maintained throughout the survey course. The informants above the age of 50–60 years were more knowledgeable. Other writers had similar findings [17,18,19,20]. Most of the participants also shared other experiences using medicinal plants. Dissemination of traditional knowledge was threatened because the younger generation had little interest in learning and had little knowledge of medicinal plants.

### 2.2. Diversity of Medicinal Plants Used

The present study reported 64 species of medicinal plants used by local people to treat dermatological ailments. A detailed description of the therapeutic flora used to treat dermatological ailments is recorded in Table 2. These plant species were known for phytochemicals like alkaloids, phenols, terpenes, flavonoids, lactins, and saponins which might explain their efficacy in treating dermatological disorders [21,22,23]. In the study area, people mostly visit the high altitudes in the summers along with their cattle, where no medical facilities are available. This is why people use these medicinal plants to treat dermatological disorders. [24] reported 111 plant species from District Kayunga Uganda, and [25,26,27] reported 25 and 36 plant species from Jammu and Kashmir, respectively.

**Figure 1:**
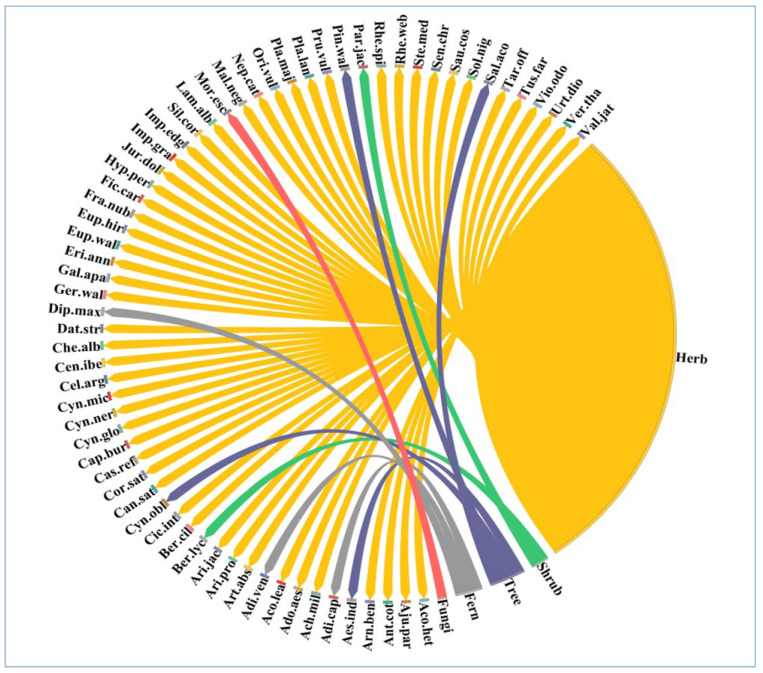
Percentage of species found among the various types of life in the study area. The direction of the lines indicates which species belong to which category of life forms, and the thickness of each bar indicates how many species are present in each category of life forms. In Table 2, each species’ full name is displayed.

The main growth habit of the used species was herbs (80%), followed by trees (8%), ferns (6%), shrubs (4%), and fungi (2%) (Figure 1). The reason behind the use of herbs might be the presence of a high content of bio-active compounds [26] and a higher efficacy than other plant parts [27]. Herbs are the primary component of forest ecosystems and are easily available in nature [28]. The plants belonged to 34 families, with Asteraceae (11 species) being the most dominant, followed by Lamiaceae (five species), Boraginaceae, and Caryophyllaceae (four species each). Other important families in the study area were Ranunculaceae (three species), Araceae, Amaranthaceae, Balsaminaceae, Pteridaceae, Euphorbiaceae, Polygonaceae, and Rosaceae (two species each), while the remaining 21 families were monotypic. The present study results aligned with previous studies where Asteraceae has been reported as the dominant medicinal plant family in various other study regions [29,30,31].

### 2.3. Plant Parts Used as Medicine

**Figure 2:**
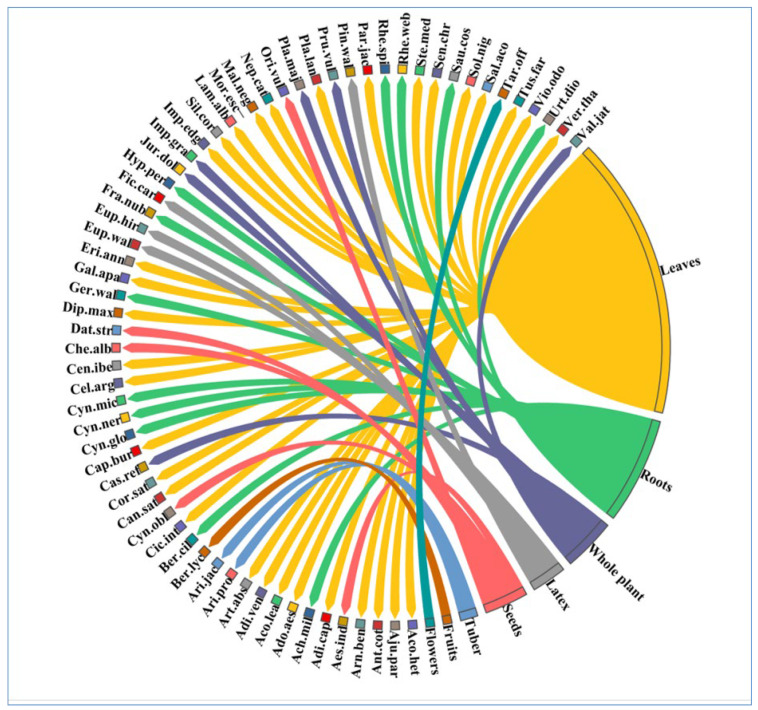
The proportion of each species found in the various plant parts used in the study area. The thickness of each bar indicates the number of species in each type of plant part used, and the direction of the lines identifies which species are related to which types of plant parts used. Table 2 displays each species’ complete name.

It was found that leaves (*n* = 32, 51%) were the most commonly used plant parts, followed by roots and the whole plant (*n* = 12, 19%), the whole plant (*n* = 6, 9%), seeds (*n* = 5, 8%), latex (*n* = 4, 6%), tuber (*n* = 2, 3%), and fruits and flowers (*n* = 1, 2% each) (Figure 2). Consistent with current research, it is reported that leaves were the main part used to cure ailments in various previous studies [32,33]. The reason behind the frequent use of leaves may be that the leaves are the center of photosynthesis and other metabolic activities, so most of the metabolites are formed in the leaves [34,35]. In addition, it is easy to collect and prepare medicines from leaves instead of other parts of the plant. It has been reported that the consumption of leaves is a sustainable collection of medicinal plants because there is no need to uproot the whole plant, so this use increases the protection of medicinal plants [36,37]. As a result of the indigenous population’s reliance on foraged plants for their daily diet, various plant sections are favored depending on how they can be used. Employing leaves and aerial components is also considered sustainable and safe [37]. Local shepherds, herbalists, and herbal drug dealers, as well as other ethnic groups, frequently prefer to utilize or trade the roots of plants for medicinal purposes [38]. Roots are also known to possess a good concentration of bioactive substances [39]. Overharvesting subsurface components or entire plants should be discouraged, especially for vulnerable species, since this practice results in eradication and a decline in the plant’s position in the wild [40,41]. Roots are often the most favored part of plants as they often comprise a higher concentration of bioactive constituents [42,43,44].

### 2.4. Mode of Preparation

**Figure 3:**
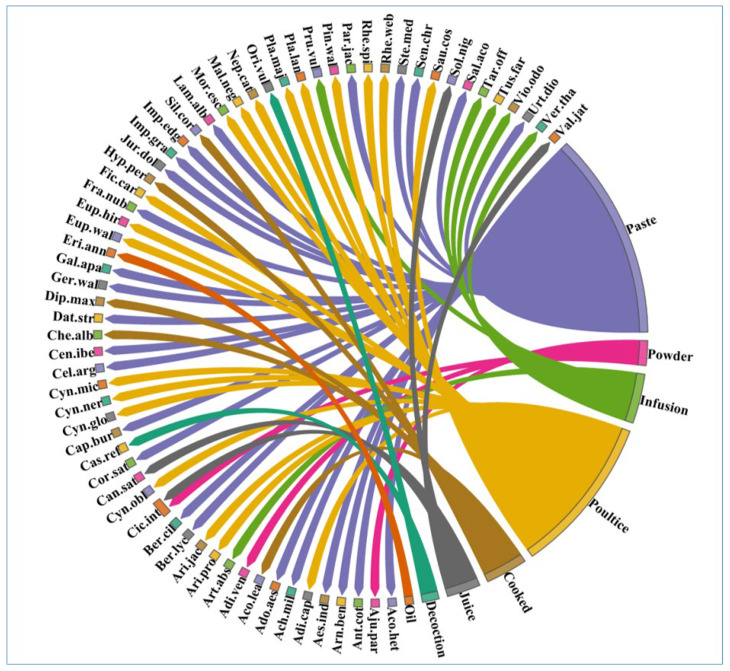
Percent of the species present in various plant preparations in the study area. The thickness of each bar indicates the number of species in each plant preparation, and the direction of the lines indicates which species are related to which type of plant preparation. Table 2 displays each species’ complete name.

The method of administration of herbal medicines used to treat dermatological ailments includes cooking, decoction, infusion, juice, paste, powder poultice, and oil. Among the various preparation methods, paste (*n* = 25, 38%) is the most common way of preparation, followed by poultice (*n* = 19, 29%), infusion (*n* = 6, 9%), cooked (*n* = 5, 8%), juice (*n* = 4, 6%), powder (*n* = 3, 5%), decoction (*n* = 2, 3%), and oil (*n* = 1, 2%) (Figure 3). Similarly, [24,45] reported paste as the most dominant preparation, consistent with our findings.

**Figure 4:**
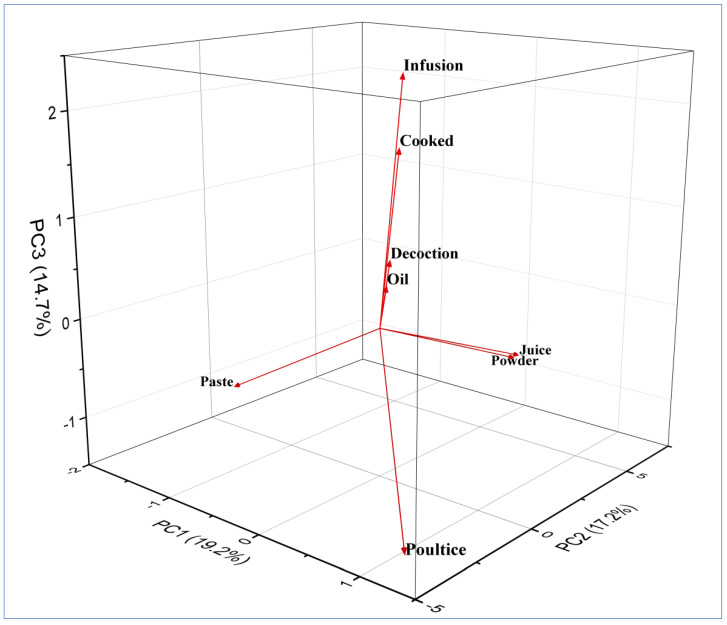
Principal component analysis (PCA) triplot of different plant preparations in district Kupwara of Jammu and Kashmir, India.

In the triplot, PC1, PC2, and PC3 explained 51.1% of the mode of preparations. Eight clusters of plant preparations based on species presence/absence can be seen here: paste, poultice, infusion, cooked, juice, powder, decoction, and oil (Figure 4). PC1 and PC2 showed a variation of 2.0, while PC1 and PC3 showed a variation of 4.5, and PC2 and PC3 showed a variation of 2.5, respectively. The plant preparations were often stored in glass bottles or other containers and used during off-seasons or during heavy snowfall in winter. According to [46], grinding, boiling, and smashing were the most common ways of extracting active ingredients in major parts of the world. Most of the plant preparations were applied topically (*n* = 54, 84%), and only (*n* = 10, 14%) were taken orally (Table 2). Our results align with [47], who reported topical application as dominant compared to oral.

### 2.5. Disease Categories in Skin Diseases and Wound Healing

**Figure 5:**
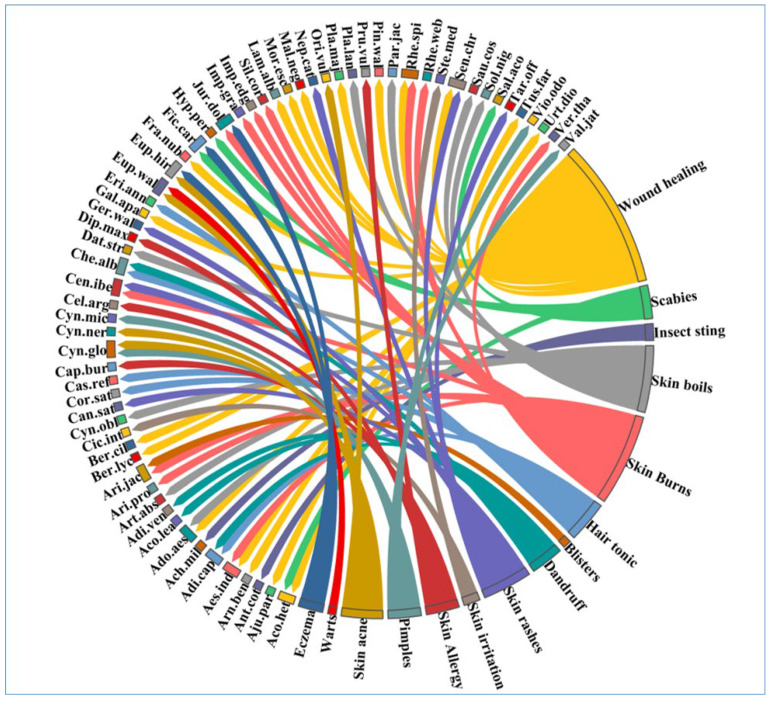
The percentage of species in the study area that fall into various disease categories. The direction of the lines indicates which species are related to which disease categories, and the thickness of each bar indicates how many species fall into each disease category. Table 2 displays each species’ complete name.

In this study, wound healing was the most dominant disease as it was cured by 18 different plant species, followed by skin burns cured by 11 plant species, skin boils (eight species), skin rashes (six species), skin acne, and hair tonic (five species each), scabies, dandruff, pimples and skin allergy (four species each), eczema (three species), insect sting and skin irritations (two species each), and warts and blisters (one species each) (Figure 5). [45,46,47] reported wound healing as the dominant disease category, which aligns with our findings. Wound healing consists of minor cuts to major injuries as most people in the study area are aligned with agricultural practices, which is the major reason for wound healing to be the dominant disease category.

**Figure 6:**
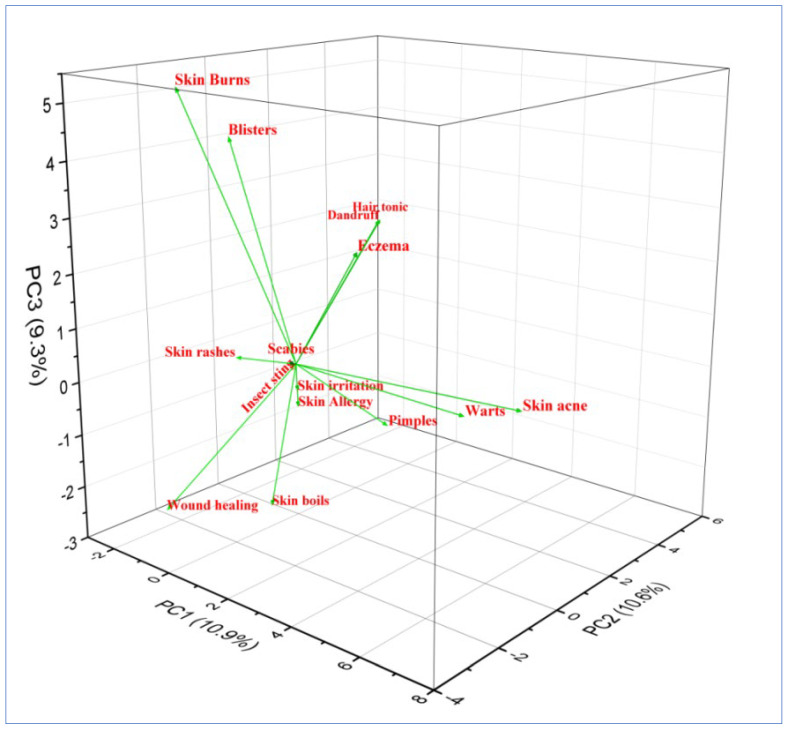
Principal component analysis (PCA) triplot of different disease categories in district Kupwara of Jammu and Kashmir, India.

In the triplot, PC1, PC2, and PC3 explained 30.8% of the disease categories. Fifteen clusters of disease categories based on species presence/absence can be seen there: wound healing, scabies, skin boils, skin burns, skin rashes, blister, hair tonic, skin acne, warts, insect sting, dandruff, skin allergy, eczema, pimples, and skin irritation (Figure 6). PC2 and PC3 showed a small variation of 0.3, while PC1 and PC2 showed a variation of 1.6, and PC1 and PC3 showed a variation of 1.3 (Figure 6). The research focuses heavily on influencing the best way to categorize diseases and treatments based on ethnomedical data, and translation from an emic to an etic outlook is necessary for the best biomedical screening of traditional treatments [48]. [49] reported wound healing as the most dominant disease category in Uttarakhand, India, which aligns with our research.

### 2.6. Medicinal Plants Used during the COVID-19 Pandemic

A total of (*n* = 20, 31.25%) plant species were used to treat different dermatological disorders during the COVID-19 pandemic. Species like *Ficus carica*, *Cichorium intybus*, *Euphorbia wallichii*, *Arisaem ajacquemontiana*, *Achillea millefolium*, *Anthemis cotula*, *Jurinea dolomiaea*, *Arnebia benthamii*, *Cynoglossum glochidiatum*, *Silene coronaria*, *Cascuta reflexa*, *Geranium wallichianum*, *Lamium alba*, *Prunella vulgaris*, *Morchella esculanta*, *Adiantum venustum*, *Pinus wallichiana*, *Rheum webbianum*, *Bergenia ciliate*, and *Verbascum thapsus* were used by different ethnic communities especially Gujjars, Bakarwals and Phari. *Ficus carica*, *Jurinea dolomiaea*, *Cichorium intybus*, *Euphorbia wallichii*, *Cynoglossum glochidiatum*, *Pinus wallichiana*, *Rheum webbianum*, and *Bergenia ciliata* were the few commonly used medicinal plants to treat different dermatological disorders.

### 2.7. Cosmetic Use of Plants

Out of 64 medicinal plants (*n* = 12, 18.75%) have cosmetic uses. Roots of *Jurinea dolomiaea*, *Rheum webbianum*, and *Rheum spiciforme* were crushed into powder and mixed with turmeric, and the paste applied topically for glowing skin. Similarly, the latex of *Ficuscarica* and *Euphorbia wallichii* were applied topically to the skin. *Coriandrum sativum*, *Adinatum capillus-veneris*, *Adiantum venustum*, and *Chenopodium album* leaves and seeds were used as a hair tonic and anti-dandruff.

### 2.8. Cross-Cultural Analysis

**Figure 7:**
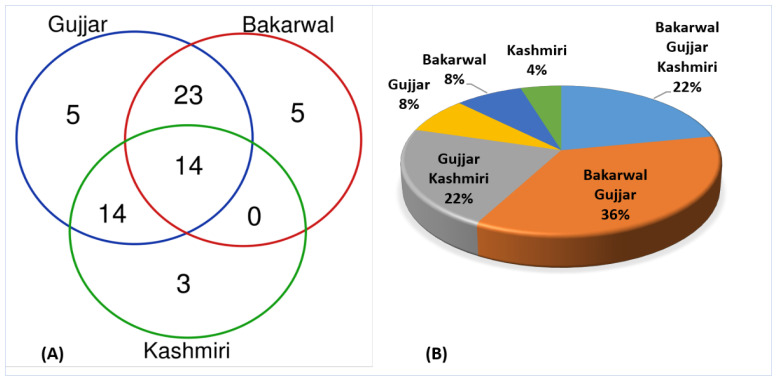
Venn diagrams (**A**), (**B**) showing a cross-culture comparison of medicinal plants used to treat different dermatological ailments among different ethnic groups.

A cross-cultural comparison of the medicinal plants used to treat dermatological ailments by the three ethnic groups has revealed a high degree of heterogeneity, and only a small proportion (14 species, 22%) of medicinal uses of certain recorded taxa were commonly shared among the three ethnic groups (Figure 7A). Between the three groups (Figure 7B), maximum homogeneity was between Gujjar and Bakarwal ethnic groups (23 species, 36%), followed by Gujjars and Kashmiri (14 species, 22%). Bakarwals and Gujjars live in the same geographical area and graze their animals in pastures, engage in extensive transhumance pastoralism, and travel through various ecological landscapes, gaining sufficient experience with certain plants and retaining more knowledge. The Bakarwal and Gujjars raise animals and have extensive traditional ecological knowledge of natural resources. They are particularly closely connected to nature due to their greater economic disadvantage and reliance on medicinal plants. The Bakarwal and Kashmiri ethnic groups show an overlap, and the dissimilarity in medicinal plants in terms of use reports may indicate certain sociocultural gaps, which in turn have prevented the sharing of traditional knowledge among the respective ethnic groups, especially since they do not intermarry (even though they share the same faith). All three groups live in the same region; therefore, geography has also played an important role in keeping the idiosyncrasy of the recorded medicinal usage among the considered groups. Comparatively, the Bakarwal ethnic and Gujjar reported maximum uses (five and eight each); the reason behind Bakarwal and Gujjar ethnic group usage is related to cattle rearing and that they mostly depend upon forest products as well as possessing more information on the medicinal uses of high mountain plant species, which is why they exclusively prefer and use these plants. The Kashmiris reported fewer plants (3, 4%), probably as a result of their urban settings and increased exposure to medical facilities and drug stores. It is significant to note that despite living in urban settings, Kashmiris reported using *Solanum nigrum*, *Anthemis cotula*, and *Cannabis sativa* in peculiar ways. This may also be related to the fact that, in contrast to the Gujjar and Bakarwal populations, which frequently intermarry, the Kashmiri community is strongly endogamous due to its geographical location. Similar results were reported by [8] from the North-West Frontier Province, Pakistan, [33,50,51] and from the Western Himalayas of Jammu and Kashmir.

### 2.9. Use Value (UV)

The use value index is used to determine the relative importance of medicinal plants in the study area. The value ranges from 0–1. Medicinal plants with the highest reports of use have high use value, while medicinal plants with few reports have the lowest use value. In the current research, the highest UV was calculated for *Ficus carica* (0.49) and the lowest UV of (0.11) for *Adonis aestivalis* (Table 2). In addition, it is not true that medicinal plants with low use values are less important, but it indicates that the knowledge of these medicinal plants is at risk or availability of the particular medicinal plant is less [23,51,52]. The high UV of medicinal plants in the study region is attributed to their common distribution in the area, and the local people were familiar with their medicinal uses [52]. The second highest value of UV is (0.42) of *Cichorium intybus* and *Euphorbia wallichii* (0.41). The highest use value of *Ficuscarica* is because it is commonly available and easy to use. Along with its dermatological uses, its fruits were edible and used to treat piles.

**Figure 8:**
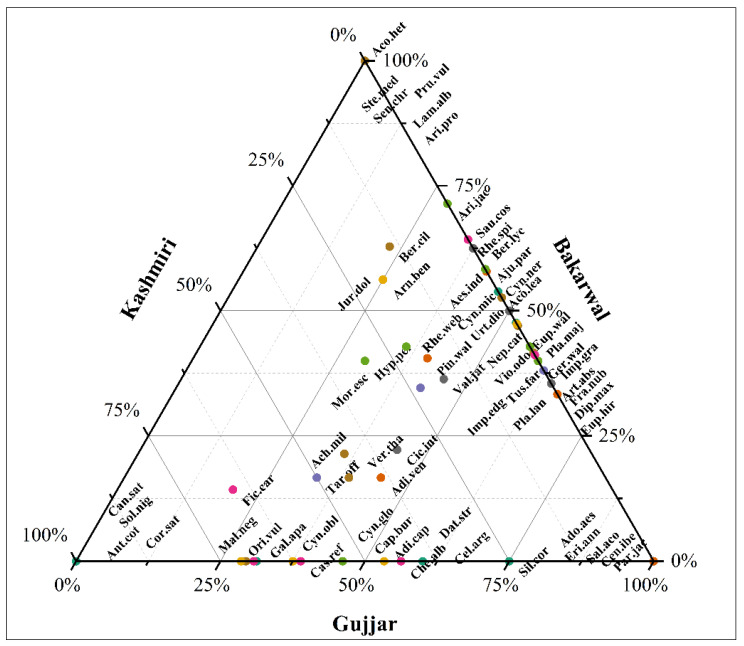
Ternary plot showing the distribution of plants and fungi on the basis of citation in different ethnic groups.

Bakarwal, followed by Gujjar, cites the highest number of plants for dermatological disorders, and the lowest number of plants is cited by the Kashmiri ethnic group (Figure 8). The reason behind this is the lack of modern medical facilities in this particular ethnic group; they commonly use traditional medicine to treat different illnesses. This particular ethnic group spent most of their time in forests (nomadic life) and transferred from one place to another, so living in a remote forest and alpine environment where modern facilities are not available means that this ethnic group is heavily dependent on traditional medicine.

### 2.10. Biological Activity and Phytochemistry of Highly Cited Plants

As a result of phytochemical studies on *Ficus carica*, phytosterols, phenolic components, organic acids, anthocyanins, amino acids, fatty acids, hydrocarbons, aliphatic alcohols, volatile components, and a few more secondary metabolites have been isolated. Other compounds exhibited antioxidant, cytotoxic, anti-cancer anti-inflammatory, and hypolipidemic actions [53]. *Ficus carica* leaf extracts in petroleum ether, chloroform, and ethanol have been shown to have anti-inflammatory properties against carrageenan-induced rat paw edema [54]. Numerous studies have focused on exploring the pharmacological properties and bioactive constituents of *F. carica* latex. The phenolic compounds present in *F. carica* latex are primarily responsible for its pharmacological activity. One such compound is mangiferin, which is abundant in *F. carica* latex extract and has several therapeutic benefits, such as anti-diabetic, anti-microbial, antioxidant, and anti-cancer effects. Additionally, *F. carica* latex is considered a valuable source of proteins, such as proteases, which are utilized to treat skin conditions such as warts and wounds [53]. Ali et al. [55] studied the antibacterial activity of *Euphorbia wallichii* against six bacteria viz., *Bacillus subtilis*, *Escherichia coli*, *Staphylococcus aureus*, *Shigella flexenari*, *Pseudomonas aeruginosa*, and *Salmonella typhi* and proved that the chloroform extract and ethyl acetate were found to be most effective exhibiting high activity. Similarly, when tested against *Staphylococcus aureus*, *Pseudomonas aeruginosa*, *Escherichia coli*, *and Candida albicans*, the crude aqueous extract, root extract, and organic seed extract of *Cichorium intybus* were found to have antibacterial activity [56]. *Euphorbia* latex has antibacterial, antioxidant, anti-inflammatory, anti-angiogenic, wound healing, cytotoxic, hemostatic, genotoxic/mutagenic, and insecticidal activities [57]. *Cichorium intybus* was studied in vivo and in vitro in mice to determine its anti-allergic activity. The results demonstrated that the aqueous extract quickly suppressed mast cell-mediated allergic responses in a dose-dependent way. In rats, it also prevented the passive cutaneous anaphylactic reaction caused by anti-dinitrophenyl IgE [58].

A wide variety of compounds, including hydrocarbons, terpene acids, organic acids, flavonoids, flavonoid glycosides, and terpene alcohols, were present in the alcoholic extracts of *Pinus wallichiana* [59], and the species showed antifungal qualities, given that the growth of *Microsporium canis* was suppressed by the n-hexane fraction of the ethanolic extract of needles at a minimum inhibitory dose of 25 L/mL [60]. *Plantago major* has shown laxative, anti-inflammatory, antipyretic, astringent, antibacterial, and immune-boosting properties. It has an immunostimulatory effect by increasing lymphocyte proliferation and interferon-gamma production [61]. *Artemisia absinthium* is regarded as a raw material for oil extraction [62]. The most frequently listed compounds are thujyl alcohol esters, α-thujone, β-thujone, camphene, α-cadinene, guaiazulene (Z)-epoxyocimene, (E)-sabinyl acetate, (Z)-chrysantenyl acetate [63]. *A. absinthium* has potent antifungal and antibacterial properties and is used in cosmetics for scalp, face, and hair care. The use of *Artemisia absinthium* in five forms is permitted by CosIng (Cosmetic Ingredient database, a European database that collects data on cosmetic ingredients). There are skincare items, perfumes, and anti-microbial compounds among them. The plant’s raw elements are applied in cosmetic items such as shampoos, face serums, masks, essences, tonics, SPF-filtered moisturizing creams, and under-eye patches. These types of cosmetics are intended to protect, cleanse, moisturize, and erase skin blemishes [64].

## 3. Materials and Methods

### 3.1. Study Area

**Figure 9:**
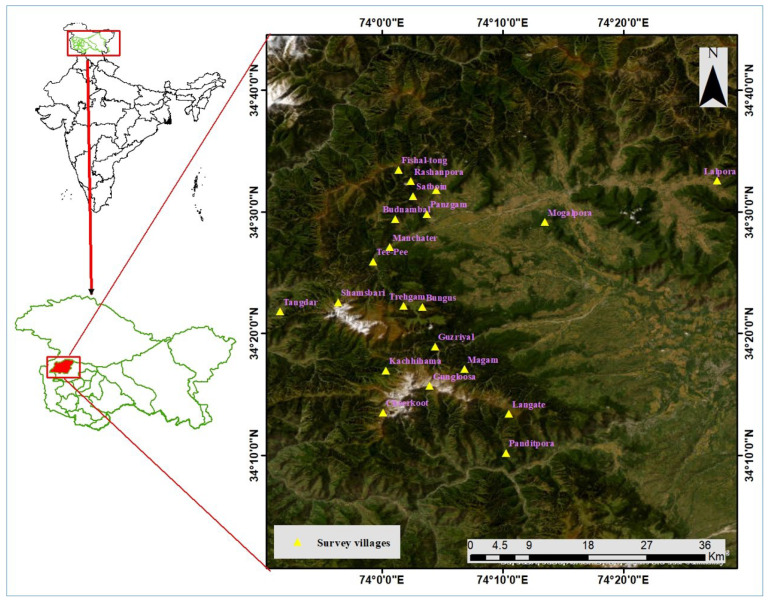
Map of the study area and points showing the surveyed villages.

The Kashmir valley of J and K (UT) has 10 districts under its jurisdiction, of which Kupwara is one of the isolated border areas, located in the northern part of Kashmir valley, at 34°01′60.00″ N and 74°15′60.00″ E. The total geographic area of the region is 2379 km^2,^ with 368 villages. According to the 2011 census, the population density is 366 persons per km^2,^ and the total population is 870,354 (Figure 9). The scheduled caste and scheduled tribe population of the area is 7.97%. The most common language spoken is Kashmiri, followed by Phari and Gojree. It is the home to many ethnic communities, such as Bakarwals (nomads occupying the high-altitude regions of Kupwara), Kashmiri (living in the main valleys and are in the majority), and Gujjars (nomad group that surrounds the area and migrates seasonally) [33,65].

The typical mountainous vegetation varies from temperate, alpine to alpine meadows, interspersed with unique taxa of shrubs and herbs. *Cedrus deodara* (Roxb. ex D.Don) G. Don, *Pinus willichiana* A.B Jacks., *Abies pindrow* (Royle ex D. Don) Royle are the main tree components of the western belts of the Himalaya. The District Kupwara includes high-altitude mountain ranges like Sadnatop (3200 m), Keran sector (1530 m–3353 m), InchasFalmarg (3048 m), RashanporaDutt (3200 m), Bungus valley (3800 m), Budnamal (2800), Raja-Ram-Ki-ladi (3500 m), while as deep valley includes Tee_Pee (2100 m), Lolab valley (1737 m). During winter, the study area faces severe cold but pleasant weather during summer. The temperature ranges between–4 °C minimum in winter and up to 32 °C maximum in summer [66].

### 3.2. Field Survey

**Figure 10:**
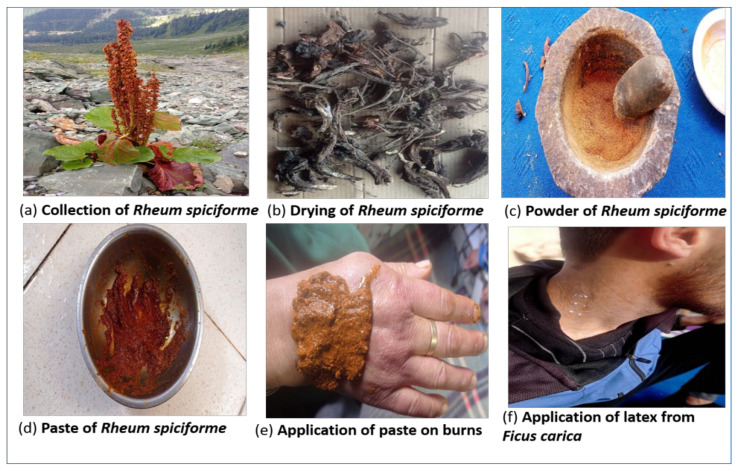
The photograph representation of *Rheum spiciforme* from (**a**) collection to (**d**) application and (**f**) application of latex from *Ficuscarica* on affected scabies.

Field trips (*n* = 35) were conducted in the spring and summer seasons of 2020–2021 to collect medicinal plants’ medicinal value and record the indigenous knowledge associated with their use. Information was gathered through open-ended semi-structured interviews (*n* = 53) and group discussions (*n* = 33) following [67]. Based on mutual agreement and in accordance with the Nagoya Protocol, neither the ethnicity of the participants nor the linguistic information provided is released. We adhered to the International Society of Ethnobiology’s code of ethics [68]. The information on ethnic medicinal plants used to treat various skin diseases like scabies (Darder), pimples (fefad), skin burns (Dazun), Acne (Dane), etc., and treatment of wounds were collected from local communities, hakims, and tribal people (Gujjar and Bakarwals). The photograph representation from the collection to the application of plants is shown in figure (Figure 10). In this study, skin diseases and wound healing were divided into 15 groups of dermatological ailments, including wound healing, dandruff, skin burns, skin boils, hair tonic, scabies, pimples, skin allergy, skin rashes, insect sting, skin acne, blisters, warts, eczema, and skin irritation. Informants were asked questions in their local language. A quantitative analysis was conducted to evaluate and organize records during the field survey. The obtained specimens were cross-checked with the assistance of a taxonomist at the University of Kashmir, Srinagar (J and K), where the specimens were also deposited. To authenticate plant names, POWO 2019 (http://www.plantsoftheworldonline.org/ accessed on 1 March 2022) was used.

### 3.3. Quantitative Data Analysis

Overall trends in the total citations and Used Value were expressed illustratively through Generalized Linear Regression Models (GLM) through Graph Pad Prism version 9 (Graph Pad Software, San Diego, CA, USA). Using the package “vegan” [69] and R software 4.0.0 [70], principal component analysis (PCA) was carried out to display the provisioning services and plant part utilized. A ternary plot was created using Origin Pro software. The circlize package in [71] R software 3.6.1 (R Core Team 2020) was used to create chord diagrams displaying the species’ percentage contributions to the study parameters (life form, plant parts used, plant preparations, and disease categories) that were applied in the study [72].

### 3.4. Use Value (UV)

The Use Value index is used to calculate the relative value of each medicinal plant species used by the local population. In the current study use value was calculated using the following formula:

UV=Ui/N

where **Ui** is the total number of use reports by each informant, and **N** indicates the total number of informants participating in the study [73]. Use Value is high when there are many usage reports for a given medicinal plant species, and use value is low when very few reports are associated with its use.

## 4. Conclusions

Medicinal plants are the backbone of our traditional healthcare system. In most developing countries, a large part of the population still depends on traditional medicine. The study attempts to highlight potential medicinal plants for the treatment of various dermatological ailments and activities of plants in the Kupwara district of Jammu and Kashmir. During the study period, 64 different medicinal plants belonging to 34 families were reported from the study area. It can be concluded from the current research that the people in the study area have a wealth of traditional knowledge inherited from their ancestors, and the record of this valuable knowledge provides new knowledge for the area. Indigenous people still depend on medicinal plants for primary healthcare but, at the same time, are alarmed by the degradation of wild flora. In order to verify this indigenous knowledge, we recommend that species with high use value (**UV**) be used for feature phytochemical and pharmacological analysis.

## Figures and Tables

**Table 1:** Demographic status of informants in the study area.

Variable	Total Percentage	Gujjar	Bakarwal	Kashmiri
Respondents	86	31 (36.05%)	26 (30.23%)	29 (33.72%)
Gender				
Male	58 (67.44%)	22 (37.00%)	16 (27.00%)	20 (34.00%)
Female	28 (32.56%)	12 (42.90%)	10 (35.60%)	6 (21.50%)
Original Language		Gojeri	Gojeri	Kashmiri
Religion		Shia and Sunni Islam	Sunni Islam	Shia and Sunni Islam
Marriages		exogamous with other Muslims (Bakarwal)	exogamous with other Muslims (Gujjar)	exogamous with other Muslims (Pahari), endogamous (Sikh)
Livelihood source		Horticulture and pastoralism	Pastoralism	Horticulture and cattle rearing
Age groups				
28–40 years	19 (22.09%)	9 (47.37%)	6 (31.58%)	4 (21.05%)
41–50 years	22 (25.58%)	7 (31.82%)	9 (40.91%)	8 (36.36%)
51–60 years	34 (39.53%)	11 (32.35%)	13 (38.24%)	10 (29.41%)
61–70+ years	11 (12.79%)	5 (45.45%)	3 (27.27%)	3 (27.27%)
Education qualification				
Illiterate	48 (55.81%)	16 (33.33%)	18 (37.50%)	14 (29.17%)
Primary	23 (26.74%)	7 (30.43%)	5 (21.74%)	11 (47.83%)
Secondary	15 (17.44%)	5 (33.33%)	3 (20.00%)	7 (46.67%)
Occupation				
Shepherds	21 (24.42%)	8 (38.10%)	10 (47.62%)	3 (14.29%)
Farmers	29 (33.72%)	10 (34.48%)	5 (17.24%)	14 (48.28%)
Hakeem’s	12 (13.95%)	4 (33.33%)	5 (41.67%)	3 (25.00%)
Housewives	15 (17.44%)	5 (33.33%)	6 (40.00%)	4 (26.67%)
Unemployed	9 (10.47%)	2 (22.22%)	4 (44.44%)	3 (33.33%)
Religion				
Islam	86 (100%)			

**Table 2:** Enumeration of plant species used by local people in district Kupwara of Jammu and Kashmir against dermatological ailments.

Family	Botanical Name and Voucher NO.	Common Name	Life Form	Part Used	Preparation	Administration	Skin Care	Total Citations	Use Value	Ethnic Groups			Native Range
										G	B	K	
Apiaceae	*Coriandrum sativum* L. (Cor.sat) 4081-KASH	Badyaan Souf	Herb	Leaves	Dried leaves are crushed into powder	Powder of leaves is mixed with mustard oil and applied topically	Hair tonic	19	0.22	----	---	19	Asia-Tropical Asia-Temperate,
Amaranthaceae	*Celosia argentea* L. (Cel.arg) 4217- KASH	Mawal, Lal jadi	Herb	Leaves	Dried leaves are crushed into powder	Powder of herb is mixed with water and applied topically	Anti-allergic	25	0.29	15	---	10	Africa
	*Chenopodium album* L. (Che.alb) 4085-KASH	Ganhaar	Herb	Seeds	Dried seeds are boiled in the water	Boiled seeds are mixed with dhesi ghee and applied topically	Dandruff and hair tonic	16	0.19	9	---	7	Asia, tropical Africa
Araceae	*Arisaema jacquemontii* Blume (Ari.jac)	Hapat Makai, Jadi booti	Herb	Tuber	Dried tuber is crushed into powder and mixed with water	A poultice of the tuber is applied topically for 2–5 days.	Skin burns	21	0.24	6	15	---	Himalayan region
	*Arisaema propinquum* Schott. (Ari.pro)	Sarfekal, Reach di jadi	Herb	Tuber	Dried tuber is crushed into powder and mixed with water	A poultice of the tuber is applied topically for 2–5 days.	Skin burns, blisters	25	0.29	----	25	---	Himalayan region
Asteraceae	*Achillea millefolium* L. (Ach.mil) 4097-KASH	Phelgass	Herb	Roots	Dried roots are crushed into powder	Dried root powder mixed with water is applied topically for 2–3 days	Insect sting	28	0.33	10	6	12	South America
	*Anthemis cotula* L. (Ant.cot)4244-KASH	Fackgass, Ganda	Herb	Leaves	Fresh leaves are crushed to make a paste	Paste is applied twice a day topically	Insect sting	15	0.17	---	---	15	Africa, Asia-Temperate, Europe
	*Artemisia absinthium* L. (Art.abs)	Tethwan, Tethyan	Herb	Leaves	Fresh leaves are soaked in water for 2 days	Infusion is taken orally early in the morning	Skin boils	31	0.36	20	11	---	Europe, Siberia, W. Himalaya
	*Centaurea iberica* Trevir. and Spreng (Cen.ibe) 4084-KASH	Kreach	Herb	Leaves	Dried leaves are crushed into a paste and mixed with water	Paste of the leaves is applied topically	Skin burns and skin rashes	16	0.19	16	---	---	Europe, W. Himalaya
	*Cichorium intybus* L. (Cic.int)	Heand, Bagh hand	Herb	Leaves	Dried leaves are boiled in water, and the extract is taken orally after cooling	Extract is taken orally early in the morning	Skin irritation	36	0.42	16	8	12	Europe, Central Asia, W. Himalaya
	*Erigeron annuus* (L.) Desf. (Eri.ann) 4087-KASH	Dargass	Herb	Seeds	Dried seeds are crushed to extract oil	Oil is used topically for 3–5 days at bedtime	Hair tonic	14	0.16	14	----	--	Europe
	*Jurinea dolomiaea* Boiss. (Jur.dol) 4091-KASH	Gogalduup, Doop	Herb	Whole plant	The dried whole plant is crushed along with water to make a paste	Paste is applied topically for 2–3 days.	Eczema and burns	32	0.37	8	18	6	W. and C. Himalayas
	*Saussurea costus* (Kuth) (Sau.cos)	Kouth, Kuth	Herb	Roots	Dried roots are crushed into powder and mixed with water	A poultice of the roots is applied topically	Boils	16	0.19	6	10	--	Central Asia, W. Himalayas
	*Senecio chrysanthemoides* DC. (Sen.chr) 4101-KASH	Bouag	Herb	Leaves	Dried leaves are crushed into powder	Paste of the leaves is applied topically	Skin rashes and wound healing	24	0.30	---	24	--	W. Himalayas
	*Taraxacum officinale* (L.) Weber ex F.H. Wigg. (Tar.off)	Saz hand, Hand	Herb	Flowers	Fresh or dried flowers are soaked in water overnight	Infusion is taken twice a day orally	Skin rashes	30	0.35	10	5	15	Europe, NW. Africa
	*Tussilago farfara* L. (Tus.far) 4103-KASH	Wat pan, Pan pati	Herb	Leaves	Fresh leaves are crushed to make a poultice	A poultice of the leaves is applied topically	Wound healing	17	0.20	10	7	---	N. Africa, W. Himalayas
Balsaminaceae	*Impatiens glandulifera* Royle (Imp.gla)	Goj-Gash, Truil	Herb	Whole plant	The whole plant is crushed into powder and mixed with water to make a paste	Paste is applied Topically for 2–3 days	Sunburns	12	0.14	7	5	---	W. Himalayas
	*Impatiens edgeworthii* Hook. f. (Imp.edg)	Truil, Tril fall	Herb	Leaves	Dried leaves are crushed into powder and mixed with water to make a paste	Paste is applied topically for 2–3 days	Skin burns	10	0.12	6	4	---	W. Himalayas
Berberidaceae	*Berberis lycium* Royle (Ber.lyc) 4102-KASH	Kawdach, Chexmachang	Shrub	Fruits	Dried fruits are crushed into powder and mixed with water to make a paste	Paste is applied topically	Healing of wounds	8	0.09	5	3	---	W. Himalayas
Brassicaceae	*Capsella bursa-pastoris* L. (Cap.bur) 4250-KASH	Kralmond	Herb	Leaves	Dried leaves are crushed into powder and mixed with mustard oil to make a paste	Paste is applied twice a day topically	Skin allergy	26	0.30	12	---	14	N. Africa
Boraginaceae	*Arnebia benthamii* Wall. ex G.Don (Arn.ben) 4096-KASH	Khazaban, Gowzaban	Herb	Leaves	Dried leaves are crushed into powder and applied directly	Powder of the leaves is applied twice a day topically	Quick healing of wounds	31	0.36	8	18	5	W. Himalayas
	*Cynoglossum glochidiatum* Wall. ex Benth. (Cyn.glo) 4083-KASH	Nil toath	Herb	Roots	Fresh roots are crushed to make a paste	Paste is applied directly on infected portions	Pimples, Skin acne	26	0.30	16	---	10	Central Asia
	*Cynoglossum nervosum* Benth. (Cyn.ner)	Nil kin, Richola	Herb	Roots	Dried roots are crushed into powder and mixed with dhesi ghee to make a poultice	Poultice is applied to affected portions twice a day	Skin acne	19	0.22	9	10	---	W. Himalayas
	*Cynoglossum microglochin* Benth. (Cyn.mic)	Cheur, Richola	Herb	Roots	Dried roots are crushed into powder and mixed with dhesi ghee to make a poultice	Poultice is applied to affected portions twice a day	Pimples	24	0.28	12	12	---	W. Himalayas
Cannabinaceae	*Cannabis sativa* L. (Can.sat)	Bang, Brand	Herb	Leaves	Dried leaves are crushed along with boiled water to make a paste	Paste is applied topically on infected portions	Skin Rashes	20	0.23	---	---	20	Central Asia, W. Himalayas
Caryophyllaceae	*Plantago major* L. (Pla.maj) 4118-KASH	Bead gull, Dada gulla	Herb	Whole plant	The fresh whole plant is crushed and applied directly	Poultice is applied on the infected portion for 2–3 days	Wound healing	34	0.40	18	16		Europe, N. and S. Africa, W. Himalayas
	*Plantago lanceolata* L. (Pla.lan)	Gull, Chota gulla	Herb	Leaves	Dried leaves are crushed along with dhesi ghee	Poultice is applied topically for 2–4 days twice a day	Skin boils	31	0.36	20	11		N. Africa, Eurasia
	*Silene coronaria* (L.) Clairv. (Sil.cor) 4229-KASH	Chock dawa	Herb	Leaves	Dried leaves are boiled in water for 10–15 min and applied after cooling	Boiled leaves are applied on affected portions for 2–3 days	Skin burns	24	0.28	18	-	6	E. Asia, Central Europe, W. Himalayas
	*Stellaria media* L. (Ste.med) 4249-KASH	Nick-haakh	Herb	Leaves	Dried leaves are crushed along with boiled water to make a paste	Paste is applied topically on infected portions	Skin irritation	11	0.13	-	11		N. Africa, Eurasia, W. Himalayas
Convolvulaceae	*Cascuta reflexa* Roxb. (Cas.ref) 4082-KASH	Kukliport	Herb	Whole plant	The dried whole plant is boiled in water for 10–20 min and taken after cooling	The decoction is taken twice a day orally	Hair tonic	17	0.20	7	-	10	Central Asia, W. Himalayas
Dryopteridaceae	*Diplazium maximum* D. Don (Dip.max)	Longued, Kunji	Fern	Young frond	Boiled and dried Young fronds are cooked as a vegetable	The cooked vegetable is eaten along with rice	Anti-allergic	15	0.17	10	5		East Asia, W. Himalayas
Euphorbiaceae	*Euphorbia wallichii* Hook. F. (Eup.wil) 4216-KASH	Hirib	Herb	Latex	The fresh stem is cut to collect the latex	Latex is applied topically for 2–3 days	Warts, Acne	42	0.49	22	20		West and Central Asia, W. Himalayas
	*Euphorbia hirta* L. (Eup.hir)	Hearib	Herb	Latex	The fresh stem is cut to collect the latex	Latex is applied topically for 2–3 days	Acne, Eczema	15	0.17	10	5		Tropical and sub-tropical America
Geraniaceae	*Geranium wallichianum* Oliv. (Ger.wal)	Ratanjog, Rati bouti	Herb	Roots	Dried roots are crushed into powder and mixed with water to make a paste	Paste is applied twice a day topically	Skin rashes	20	0.23	12	8		W. Himalayas, Central Asia
Hamamelidaceae	*Parrotiopsis jacquemontiana* (Decne.) Rehder. (Par.jac)	Poah	Shrub	Leaves	Fresh leaves are crushed to make a paste	Paste is applied twice a day topically	Skin Boils	15	0.17	15	-		W. Himalayas, South Asia
Hypericaceae	*Hypericum perforatum* L. (Hyp.per) 4090-KASH	Chai kul, Julab ki jadi	Herb	Roots	Fresh roots are boiled in water for 10–15 min, and the extract is taken after cooling	The decoction is taken orally early in the morning for one week	Scabies	10	0.12	3	4	3	NW Africa, W. Himalayas, Europe
Lamiaceae	*Ajuga parviflora* Benth (Aju.par)	Jaind, Javind	Herb	Leaves	Dried leaves are crushed to make a powder	Powder is applied topically on affected portions	Wound healing	19	0.22	11	8		W. Himalayas, South Asia
	*Lamium album* L. (Lam.alb) 4092-KASH	Zakhmedawa	Herb	Leaves	Fresh leaves are crushed to make a paste	Paste is applied topically on affected portions	Wound healing	25	0.29	---	25		Eurasia, W. Himalayas
	*Nepeta cataria* L. (Nep.cat) 4093-KASH	Gandsoi	Herb	Leaves	Dried leaves are crushed into a powder and mixed with mustard oil	Poultice is applied topically on affected portions	Skin irritation	15	0.17	9	---	6	E Europe, W. Himalayas
	*Origanum vulgare* L. (Ori.vul) 4100-KASH	Baber, Baberi	Herb	Seeds	Dried seeds are boiled in water for 15–30 min to make a decoction	The decoction is taken twice a day orally	Acne	28	0.33	8	--	20	Europe, Africa, W. Himalayas
	*Prunella vulgaris* L. (Pru.vul)	Kalyuth, Singrota	Herb	Whole plant	The fresh whole plant is soaked in water overnight to make an infusion	infusion is taken twice a day orally for 2–3 days	Skin allergy	31	0.36	---	31		Europe, Asia, W. Himalayas
Malvaceae	*Malva sylvestris* L. (Mal.syl)	Sochal, sucheal	Herb	Leaves	Fresh leaves are crushed to make a poultice	Poultice is applied topically on affected portions	Wound healing	24	0.28	8	---	18	Europe, C Asia, W. Himalayas
Morchellaceae	*Morchella esculenta* Fr. (Mor.esc) 4215-KASH	Gucchii	Fungi	Fruiting body	Dried fruiting body is crushed into powder and mixed with water or walnut oil to make a poultice	Poultice is applied topically on affected portions	Wound healing	26	0.30	11	9	6	W. Himalayas
Moraceae	*Ficus carica* L. (Fic.car) 4089-KASH	Anjeer	Tree	Latex	Leaves are cut to collect the latex	Latex is applied topically for 2–3 days on affected portions	Scabies, Eczema	35	0.41	7	5	23	Central Asia, W. Himalayas
Pteridaceae	*Adinatum capillus-veneris* L. (Adi.cap) 4115-KASH	Guether	Fern	Leaves	dried leaves are crushed into a powder and mixed with mustard oil or dhesi ghee	The mixture is applied topically at bedtime for 2–3 days	Hair tonic and Dandruff	15	0.17	8	----	7	Europe
	*Adiantum venustum* D. Don (Adi.ven) 4104-KASH	Guetheer	Fern	Leaves	dried leaves are crushed into a powder and mixed with mustard oil or dhesi ghee	The mixture is applied topically at bedtime for 2–3 days	Dandruff	18	0.21	8	3	7	W. Himalayas
Pinaceae	*Pinus wallichiana* A. B. Jacks (Pin.wal) 4227-KASH	Kayur, Fir	Tree	Latex	Latex is collected by making a deep cut into the stem of the tree using an axe	Latex is mixed with salt and applied topically on affected portions	Wound healing	37	0.43	15	15	7	W. Himalayas
Polygonaceae	*Rheum spiciforme* Royle (Rhe.spi)	Chithola, pambchalan	Herb	Roots	Dried roots are crushed into powder and mixed with water to make a poultice	Poultice is applied topically for 2–3 days	Wound healing and skin burns	24	0.28	5	19	----	W. Himalayas
	*Rheum webbianum* Royle (Rhe.web) 4212-KASH	Chithola, pambchalan	Herb	Whole plant	Dried roots are crushed into powder and mixed with water to make a poultice	Poultice is applied topically for 2–3 days	Skin burns and wound healing	28	0.33	10	12	6	W. Himalayas
Ranunculaceae	*Aconitum heterophyllum* Wall. ex Royle (Aco.het) 4049-KASH	Patris, itees	Herb	Leaves	Fresh leaves are crushed into a paste	Paste is applied topically on affected portions	Scabies and healing of wounds.	14	0.16	---	14	---	W. Himalayas
	*Adonis aestivalis* L. (Ado.aes)	Tankbutton	Herb	Leaves	Fresh leaves are crushed into a paste	Paste is applied topically on affected portions	Skin boils and healing of wounds.	9	0.11	9	--	--	Europe
	*Aconitum leave* Royle (Aco.lea)	Mori	Herb	Leaves	Fresh leaves are boiled in water for 5–10 min	Boiled leaves are applied topically on affected portions	Dandruff	13	0.15	6	7	--	W. Himalayas
Rosaceae	*Cyndonia oblonga* Mill. (Cyn.obl)	Bumbchont	Tree	Seeds	Dried seeds are crushed into powder and mixed with dhesi ghee to make a poultice	Poultice is applied topically on affected portions for 2–3 days	Skin boils	16	0.17	5	---	11	Europe, Central Asia, W. Himalayas
	*Fragaria nubicola* Lindl. Ex Lacaita (Fra.nub) 4088-KASH	Ringrish, jungle gounch	Herb	Whole plant	The fresh whole plant is washed in water and crushed to make a paste	Paste is applied topically on affected portions	Wound healing	19	0.22	13	8	--	Central Asia, W. Himalayas
Rubiaceae	*Galium aparine* L. (Gal.apa)	Thapgass	Herb	Whole plant	The fresh whole plant is washed in water and crushed to make a paste	Paste is applied topically on affected portions twice a day	Wound healing	18	0.21	5	---	12	Eurasia
Saxifragaceae	*Bergenia ciliata* (Haw.) Sternb. (Ber.cil) 4213-KASH	Palfort, Butpewa	Herb	Roots	Dried leaves crushed into powder and mixed with water to make a paste	Paste is applied Topically on affected portions	Healing of wounds	35	0.41	8	22	5	W. Himalayas
Sapindaceae	*Aesculus indica* (Wall. ex Cambess.) Hook. (Aes.ind) 4111-KASH	Haandoon	Tree	Seeds	Seeds are crushed into powder and mixed with water to make a paste	Paste is applied Topically on affected portions	Skin burns and wounds	12	0.14	5	7	--	W. Himalayas
Salicaceae	*Salix acomphylla* Boiss. (Sal.aco)	Veer kul, veer	Tree	Leaves	Fresh leaves are crushed to make a paste	Paste is applied Topically on affected portions	Scabies	13	0.15	13	---	--	Europe, W. Himalayas
Scrophulariaceae	*Verbascum thapsus* L. (Ver.tha) 4242-KASH	Sarfe Makai, Bunder tund	Herb	Leaves	Fresh leaves are soaked in water for 2 days to make an infusion	Infusion is taken orally for 2–4 days twice a day	Skin burns	18	0.21	7	3	8	Europe, W. Himalayas
Solanaceae	*Datura stramonium* L. (Dat.str) 4086-KASH	Datur, Datuer	Herb	Seeds	Dried seeds are crushed into powder and mixed with water to make a paste	Paste is applied topically on affected portions	Skin boils	16	0.19	9	---	7	C. America
	*Solanum nigrum* L. (Sol.nig)	Kambailkul	Herb	Leaves	Dried leaves are crushed into powder and mixed with water to make a paste	Paste is applied topically on affected portions	Skin boils	21	0.24	---	---	21	Eurasia, N. and E. Africa, W. Himalayas
Urticaceae	*Urtica dioica* L. (Urt.dio) 4219-KASH	Soi	Herb	Leaves	Dried leaves are crushed into powder and mixed with water to make a paste	Paste is applied topically on affected portions	Wound healing	19	0.22	10	9	---	Europe, N. and W. Africa, W. Himalayas
Valerianaceae	*Valeriana jatamansi* Jones (Val.jat) 4237-KASH	Mushkbalay	Herb	Whole plant	The fresh whole plant is crushed to make a paste	Paste is applied topically on affected portions	Pimples	22	0.26	10	8	4	Central Asia, W. Himalayas
Violaceae	*Viola odorata* L. (Vio.odo)	Banposh, Vanpoash	Herb	Roots	Dried roots are crushed to make a poultice	Poultice is applied topically on affected portions	Pimples	14	0.16	8	6	---	Europe, N. and W. Africa

## Data Availability

All data have already been included in the manuscript.
